# IFI30 expression predicts patient prognosis in breast cancer and dictates breast cancer cells proliferation via regulating autophagy

**DOI:** 10.7150/ijms.62870

**Published:** 2021-07-25

**Authors:** Yan Fan, Xu Wang, Yang Li

**Affiliations:** 1 Department of Pediatrics, The First Affiliated Hospital of China Medical University, No. 155, North Nanjing Street, Heping District, Shenyang , Liaoning, 110001 China.; 2 Department of Breast Surgery, Department of Surgical Oncology, Research Unit of General Surgery, The First Affiliated Hospital of China Medical University, No. 155, North Nanjing Street, Heping District, Shenyang , Liaoning, 110001 China.; 3 Department of Cell Biology, Key Laboratory of Cell Biology, National Health Commission of the PRC, and Key Laboratory of Medical Cell Biology, Ministry of Education of the PRC, China Medical University, No. 77, Puhe Road, Shenyang North New Area, Shenyang, Liaoning,110122 China.

**Keywords:** Interferon γ-inducible protein 30 (IFI30), breast cancer, cell proliferation, autophagy

## Abstract

**Introduction**: Incidence and mortality rates of breast cancer are increasing in women worldwide. Immunotherapy is a relatively popular treatment modality for all malignant tumors including breast cancer in recent years. Interferon γ-inducible protein 30 (IFI30) could catalyze the reduction of disulfide bonds and enhance major histocompatibility complex (MHC) class II-restricted antigen processing. Recent studies showed that IFI30 played an important role in the immune response of malignant tumors.

**Methods**: The Cancer Genome Atlas (TCGA) database and clinical proteomic tumor Analysis consortium (CPTAC) database were applied to predict the role of IFI30 in breast cancer and the relationship between IFI30 and prognosis of breast cancer patients. Then we detected the expression of IFI30 in clinical samples of breast cancer patients, and analyzed the relationship between IFI30 and the prognosis of breast cancer patients. We used lentivirus infection method to construct a stable IFI30 knockdown cell line, and detected the effect of IFI30 in breast cancer cells. Nude mice tumor bearing experiment was performed to investigate the effect of IFI30 on breast cancer cells *in vivo*. Western blot was used to verify the regulation of autophagy related protein LC3 and p62 by IFI30.

**Results:** We found that IFI30 was highly expressed in breast cancer tissues and was associated with poor outcome of patients. The knockdown of IFI30 could inhibit the proliferation, migration and invasion of breast cancer cells and significantly inhibit tumor growth *in vivo*. Increased accumulation of LC3-II and p62 suggested impaired autophagy in IFI30 knockdown cells.

**Discussion:** As a result, we suggested that IFI30 might play a key role in the initiation and progression of human breast cancer and might be a new therapeutic target in breast cancer.

## Introduction

Breast cancer is the most common malignant tumor in women worldwide and the main cause of death in women due to malignant tumors. In recent years, the incidence of breast cancer in women has risen sharply 1, and the burden of disease is also increasing. Therefore, early diagnosis and treatment of breast cancer are particularly important. Breast cancer is highly heterogeneous. More and more attention has been paid to the individualized treatment of breast cancer, so it is important to search for new diagnostic markers and molecular targets for breast cancer treatment.

At present, the main treatments for breast cancer are surgery, radiotherapy, chemotherapy, endocrine therapy and targeted therapy, while immunotherapy is a relatively popular treatment modality for all malignant tumors including breast cancer in recent years. In clinical treatment of triple negative breast cancer, programmed cell death protein 1(PD1) monoclonal antibody combined with albumin and paclitaxel has achieved good results in the treatment of breast cancer 2.

Interferon γ-inducible protein 30 (IFI30), also known as gamma-interferon-inducible lysosomal thiol reductase (GILT), is a reductase localized in endosomes, lysosomes and phagosomes 3. IFI30 is constitutively expressed in most antigen presenting cells (APCs), including monocytes/macrophages, B cells (primary and cell lines), and bone-marrow derived dendritic cells (DCs) 4-9. IFI30 is also constitutively expressed in thymocytes 10, mature T cells 10, 11, and some fibroblasts 12, 13. Human IFI30 is composed of 261 amino acids with a 37 amino acid signal sequence and a 224 amino acid precursor form. The 35 kDa precursor is tagged with mannose-6-phosphate (M6P) residues and targeted to the endocytic pathway via the M6P receptor (M6PR) 4, 9. The most well described function of IFI30 is considered enhancing MHC class II-restricted antigen processing 14. As a reductase, IFI30 could catalyze the reduction of disulfide bonds that is an important step in MHC class II-restricted processing and presentation in antigens. It generates cell surface MHC class II peptide complexes which is essential for the activation of CD4^+^ T lymphocytes 3, 15.

Recent researches have revealed that IFI30 plays an indispensable role in the immune response of malignant tumors, such as melanoma, prostate cancer and glioma 16-18. However, the role of IFI30 in breast cancer is still poorly understood. In this study, the effect of IFI30 on breast cancer was investigated. TCGA and CPTAC database analysis showed that IFI30 mRNA and protein were highly expressed in breast cancer tissues, and the high expression of IFI30 mRNA was associated with poor outcome of patients. Furthermore, the high expression of IFI30 protein was also confirmed in clinical fresh samples and wax lumps, which was related to the poor overall survival (OS) and distant metastasis-free survival (DMFS) of patients. In addition, lentivirus mediated small hairpin RNA (shRNA) was used to knock down IFI30 gene to explore its function in breast cancer cells. Knockdown of IFI30 could significantly inhibit the proliferation, colony formation, migration and invasion of breast cancer cells. *In vivo*, tumor was significantly inhibited in nude mice. Increased accumulation of LC3-II and p62 suggested impaired autophagy in IFI30 stable knockdown MDA-MB-231 and SK-BR-3 breast cancer cells.

## Materials and Methods

### Clinical samples and mice

60 paired breast cancer tissues and paracancerous normal tissues (5 cm from the edge of the tumor) and Paraffin-embedded sections of tissue specimens (n=82, between 2008 and 2009, supplementary Table S1) were collected from the Department of Breast Surgery of the First Affiliated Hospital of China Medical University. All patients were pathologically diagnosed as breast cancer distinctly on the basis of the American Joint Committee Cancer (AJCC) 19. After resection, fresh tumor tissues and adjacent normal tissues were frozen in liquid nitrogen immediately and then stored at - 80°C. The study was approved by the Committee on Medical Ethics and Human Clinical Trials of China Medical University. All participants have agreed and signed an informed consent. This study was conducted in accordance with the Declaration of Helsinki. Female BALB/C nude mice aged 5-6 weeks were purchased from Vital River Laboratory Animal Technology Co., Ltd (Beijing, China). All of the animal experiments were carried out in accordance with the Guidelines for the Care and Use of Laboratory Animals (NIH publication no. 80-23, revised 1996) approved by the Animal Ethics Committee of China Medical University.

### Cell culture

Human breast cancer cell line MDA-MB-231 was cultured in Leibovitz's L-15 (Thermo Fisher Scientific Inc. United States. Gibco. 11415064) containing 10% fetal bovine serum (FBS) (Thermo Fisher Scientific Inc. United States. Gibco. 10099141) and human breast cancer cell line SK-BR-3 was cultured in McCoy's 5a modified medium (Thermo Fisher Scientific Inc. United States. 16600082) containing 10% FBS. All the cells were incubated in a humidified incubator containing 5% CO_2_ at 37°C.

### Lentiviral infection

IFI30 short hairpin RNAs (IFI30-shRNA) and control lentivirus were obtained from Shanghai Genechem Co., Ltd. The IFI30-shRNA 1# sequence was 5′-ccAGACACTATCATGGAGTGT-3′; the IFI30-shRNA 2# sequence was 5′-ccCTACGGAAACGCACAGGAA-3′; the IFI30-shRNA 3# sequence was 5′-gcGTTAGACTTCTTTGGGAAT-3′ and the shRNA control sequence was 5′-TTCTCCGAACGTGTCACGT-3′. MDA-MB-231 cells and SK-BR-3 cells were seeded into 12-well plates overnight. Then, according to the manufacturer's guidelines (GeneChem, China), the seeded cells were infected with IFI30-shRNA 1#, IFI30-shRNA 2#, IFI30-shRNA 3# and control lentivirus. To select infected cells, 5 μg/ml puromycin (Merck KGaA, Darmstadt, Germany. Sigma-Aldrich LLC. 54022) was added to the medium and the lentiviral infection rate was detected by fluorescence microscopy 5 days after infection.

### Western blot assay

Tissues and cells were lysed in a radio immunoprecipitation assay (RIPA) lysis buffer which was added with protease inhibitors (Merck KGaA, Darmstadt, Germany. Roche Molecular Systems. 11697498001). After protein extraction, the protein concentration was determined. Total protein extracts were separated by 8% sodium dodecyl sulfate-polyacrylamide gel electrophoresis (SDS-PAGE) and then transferred to the polyvinylidenefluoride (PVDF) membranes (Merck KGaA, Darmstadt, Germany. Millipore. IPVH00010). After transferring, the PVDF membranes were dyed with Ponceau red and cut according to the molecular weight of protein. PVDF membranes were blocked in 5% skimmed milk for 1 hour and incubated with corresponding anti-IFI30 (1: 1000; proteintech, China, 11597-1-AP), anti-LC3 (1: 1000; Abcam plc. England. ab192890), anti-p62 (1: 1000; proteintech, China. 18420-1-AP), anti-Actin (1:5000; Abcam plc. England, as a loading control) antibodies at 4°C overnight. After washing the PVDF membranes with Tris Buffered saline Tween (TBST) and adding the correct anti-mouse or anti-rabbit antibodies, the PVDF membranes were incubated on the shaker at room temperature for 2 hours. Finally, an enhanced chemiluminescence (ECL) system (Thermo Fisher Scientific Inc. United States. Thermo pierce. 32209) was used to detect the target proteins.

### Transwell migration and invasion assay

Cell suspension was prepared and the cell density was adjusted to 1×10^5^ /ml. In order to detect the migration and invasion ability of the cells, 100μl shIFI30 and Con lentivirus-infected MDA-MB-231 cells were seeded into the upper transwell chambers separately (8-μm BioCoat Control Inserts, Corning Costar, United States. 3422). The lower chamber was filled with 600 μl Leibovitz's L-15 supplemented with 10% FBS. Cells in the chambers were incubated for 24 hours at 37°C. After wiping off the matrix glue and the cells in the upper chambers with a cotton swab, the cells in the lower chambers were fixed by 95% alcohol and stained with 0.4% trypan blue. Cells were counted (five random fields per well at 100× magnification) under a light microscope. For invasion assay, 5 × 10^4^ cells were seeded in the matrigel-coated upper chamber (Corning. United States. 356234) and the migration assay was performed as above.

### CCK8 proliferation assay

Cell suspension was prepared. ShIFI30 and nonspecific control (NC) stable transfected MDA-MB-231 cells were seeded in 96-well plates (3 × 10^3^) for cell viability assay. CCK8 was added at 1, 2, 3, 4 and 5 day of cell attachment, incubated at 37 ° for 2 hours, and detected by enzyme labeling instrument. Respectively. The absorbance of each sample was measured at 490 nm and gained the optical density (OD) values.

### Colony formation assay

Each cell line was seeded in triplicates wells at a density of 200 cells/well in 6-well plates. Cells treated with shRNA were kept under puromycin selection. The cells were incubated in a saturated humidified incubator containing 5% CO_2_ at 37°C for 14 days. After 14 days, the cells were fixed with ice cold methanol for 15 minutes and stained with Trypan Blue for 10-30 minutes. The cells were photographed under a microscope (Leica, Buffalo Grove, IL). Number of colonies per well and colony size were quantified using Image J.

### Immunohistochemistry

After deparaffinization and hydration, tumor sections were incubated in 3% hydrogen peroxide in methanol to eliminate endogenous peroxidase activity, and after antigen retrieval, incubated with normal serum. Sections were incubated with primary antibody IFI30 (1: 200; proteintech, China. 11597-1-AP) overnight at 4°C. Followed by incubation with secondary antibodies, sections were stained with diaminobenzidine (DAB; Zhongshan Biotechnology Company, China. ZLI-9017), counterstained with hematoxylin (Maixin Biotechnology Co., Ltd. China. CTS-1099), differentiated in hydrochloric acid alcohol. After hydration and transparency, the sections were sealed with neutral resins and photographed.

### Tumor-bearing in nude mice

The MDA-MB-231 cells with stable knockdown of IFI30 and control MDA-MB-231 cells were trypsinized, washed with phosphate buffer solution (PBS) once, and resuspended in PBS (2 × 10^6^/ml). 100 micro liters of cell suspension was injected subcutaneously into the left axilla of BALB/c nude mice (n = 5 per group). Measurements started after tumors were palpable at 28 days after injection. The tumor size of each mouse was measured every 3 days. The mice were anesthetized and then euthanized 40 days after injection. Tumors were extracted and washed in PBS, then fixed in 4% paraformaldehyde.

### Statistical analysis

Statistical analyses were conducted using SPSS 20.0 (Chicago, IL, USA) and GraphPad Prism 8.0 software. Student's t-test (2-tailed) was applied to compare differences between two groups in repeated studies. The significance of differences in specimen data was determined with a chi-square test. The Kaplan-Meier method was used to generate survival curves. *p <0.05, ** p <0.01, *** p <0.001 and **** p <0.0001 were considered statistically significant.

## Results

### Database analysis showed that the expression of IFI30 mRNA and protein in breast cancer tissues were significantly higher than those in normal tissues

**Figure 1:**
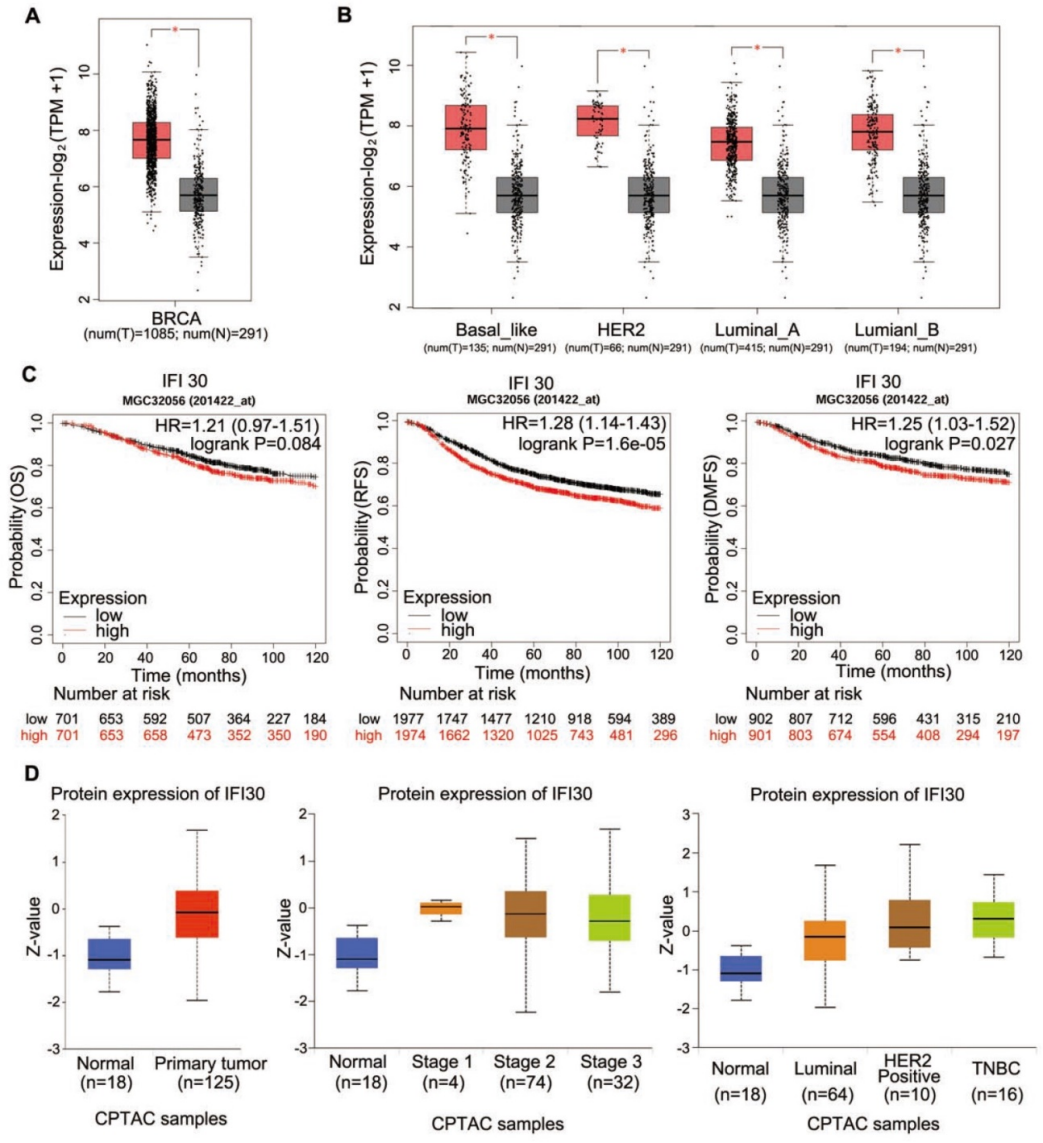
Database analysis showed that the expression of IFI30 mRNA and protein in breast cancer tissues were significantly higher than those in normal tissues. (A) Predict the mRNA expression of IFI30 in breast cancer and adjacent cancer in TCGA database. (B) Predict the mRNA expression of IFI30 in different molecular types of breast cancer and adjacent cancer in TCGA database. (C) Predict the correlation of the overall survival (OS), recurrence free survival (RFS) and distant metastasis-free survival (DMFS) and IFI30 mRNA level in TCGA database. (D) Predict the protein expression of IFI30 in breast cancer and adjacent cancer in CPTAC database. Left: In breast cancer and adjacent cancer. Middle: In different stages of breast cancer and adjacent cancer. Right: In different molecular types of breast cancer and adjacent cancer.

As IFI30 could affect the tumorigenesis and development of cancers, we analyzed TCGA database to detect the expression of IFI30 mRNA in breast cancer tissues and found that the expression level of IFI30 mRNA in breast cancer tissues was higher than that in normal tissues obviously (Fig. 1A). Molecular typing of breast cancer plays an important role in guiding clinical treatment and judging prognosis. In order to further understand the expression of IFI30 mRNA in different molecular types of breast cancer, we continued to analyze according to the molecular typing of breast cancer in TCGA database. The results showed that IFI30 mRNA is all highly expressed in basal like, human epidermal growth factor receptor 2 (HER-2) positive, Luminal A and Luminal B molecular types of breast cancer (Fig. 1B). Next, according to TCGA database, the correlation between IFI30 mRNA expression and prognosis of breast cancer was analyzed. It was found that the high expression of IFI30 mRNA was associated with poor OS, recurrence free survival (RFS) and DMFS (Fig. 1C). The results above suggested that high expression of IFI30 mRNA was associated with poor prognosis of breast cancer.

Protein is the executor of gene's final function; therefore, we used bioinformatics to analyze the expression of IFI30 protein in breast cancer. CPTAC database was used to analyze the IFI30 protein expression (Fig. 1D). We found that IFI30 protein expression in breast cancer was significantly higher than that in normal tissues, especially in triple negative breast cancer.

### High expression of IFI30 protein was related to metastasis and prognosis of breast cancer

**Figure 2:**
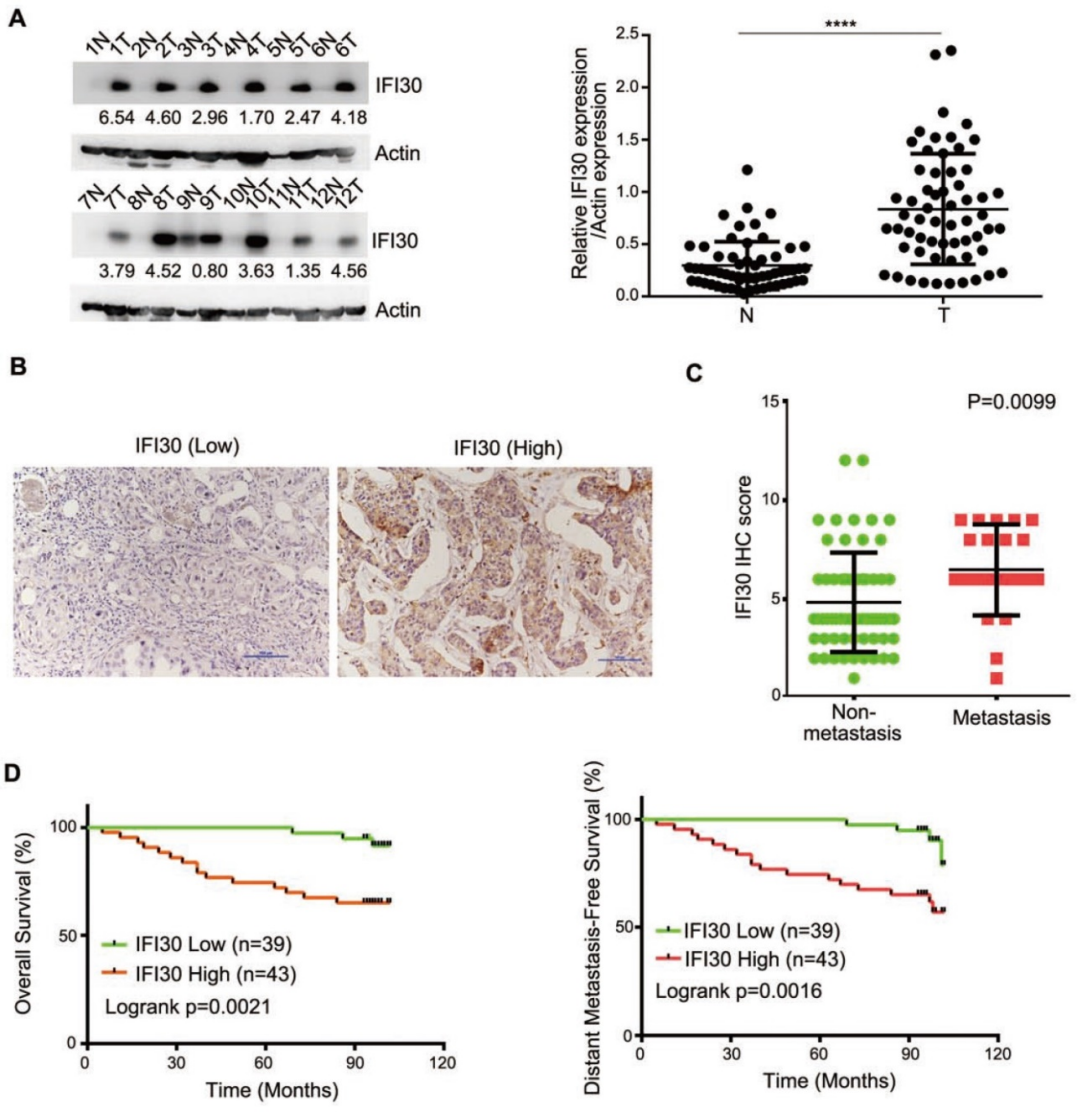
High expression of IFI30 protein was related to metastasis and prognosis of breast cancer. (A) IFI30 protein expression in 60 clinical breast tissue pairs. Lysates of tumor tissues (T) and matched adjacent noncancerous tissues (N) were analyzed using Western blot assay. Twelve representative pairs are shown. Right: The indicated protein levels in left were statistically analyzed. (****p < 0.0001). (B) Representative images of immunohistochemical staining from two cases. Original magnification, 200×. (C) High IFI30 expression associates with metastasis of breast cancer. The subjects with breast cancer were divided into two groups based on non-metastasis (n=61) and metastasis (n=21). The intensity values in (B) are expressed as the H-score. The middle, upper, and lower lines in the boxplot represent the mean and the upper and lower quartiles, respectively. (D) High IFI30 expression associates with poor clinical outcomes of breast cancer. Kaplan-Meier survival analyzed (GraphPad) the relationship between overall survival (upper) and disease-free survival (lower) in breast cancer cases and IFI30 expression. P value was calculated by log-rank test.

According to the bioinformatics prediction, we detected the expression of IFI30 protein in clinical breast cancer samples as well as corresponding non-tumor normal tissues to determine the role of IFI30 in breast cancer. We performed Western blot assay on fresh samples from 60 pairs of breast and adjacent noncancerous tissues and found that IFI30 protein was highly expressed in breast cancer tissues (Fig. 2A). Immunohistochemistry was applied to assess the association with clinicopathological characteristics and the outcomes of patients. Immunohistochemistry results in 82 cases of breast cancer tissue wax blocks suggested that IFI30 was high expressed in breast cancer. Representative images are shown (Fig. 2B). Histopathologic analyses of 82 breast cancer specimens revealed that higher IFI30 expression was significantly associated with distant tumor metastasis (Table 1). Immunohistochemistry results were further analyzed and the results obtained showed that high IFI30 expression correlated with breast cancer metastasis and poor OS and DMFS in breast cancer patients (Fig. 2C and Fig. 2D). To evaluate the impact of IFI30 and pathological factors on the prognosis of the breast cancer patients, we performed univariate and multivariate analyses using the Cox proportional hazards model. The univariate analysis of the OS rate revealed statistically significant variable: the overexpression of IFI30 (P=0.006). In the multivariate analyses, IFI30 (P=0.006) was associated with a poor OS rate (Table 2). The above experimental results further confirmed that IFI30 expression increased in breast cancer tissues, which was correlated with poor patient prognosis.

### Knockdown of IFI30 could inhibit the proliferation, migration and invasion of breast cancer cells

**Figure 3:**
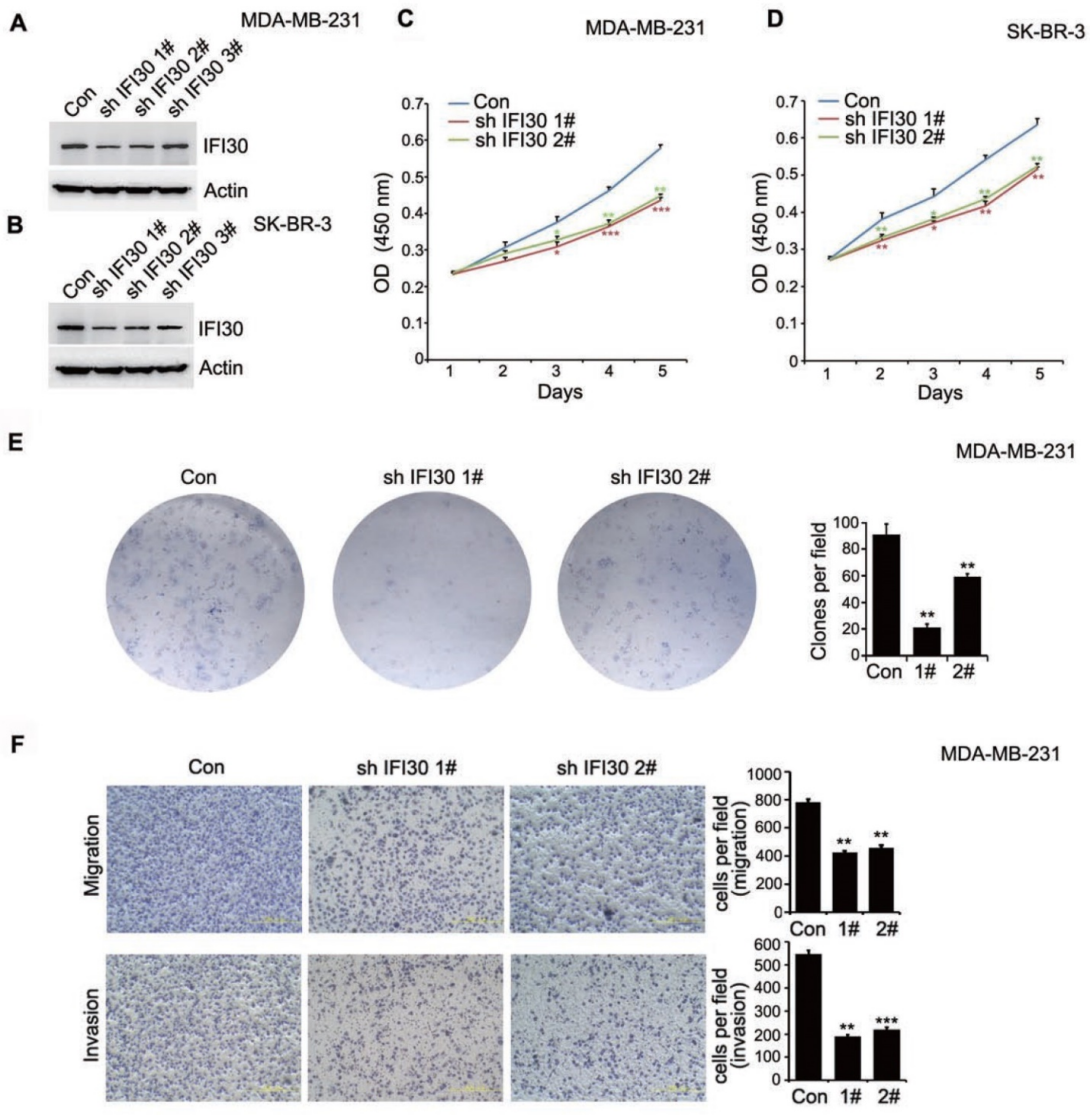
Knockdown of IFI30 could inhibit the proliferation, migration and invasion of breast cancer cells. (A) IFI30 protein expression in MDA-MB-231 cells after transfection of shIFI30 or shCtrl (Con) lentiviruses. Actin was used as an internal control. (B) IFI30 protein expression in SK-BR-3 cells after transfection of shIFI30 or shCtrl (Con) lentiviruses. Actin was used as an internal control. (C) The proliferation of MDA-MB-231 cells after IFI30 knockdown. CCK8 (Cell Counting Kit 8) was added at 1, 2, 3, 4 and 5 day of cell attachment in MDA-MB-231cells, incubated at 37°C for 2 hours, and detected by enzyme labeling instrument. The data are presented as a histogram of the mean _ SEM of three independent experiments (* p < 0.05, ** p<0.01, *** p<0.001, n =3). (D) The proliferation of SK-BR-3 cells after IFI30 knockdown. The data are presented as a histogram of the mean _ SEM of three independent experiments (* p < 0.05, ** p<0.01, n =3). (E) IFI30 knockdown inhibits colony formation. Representative pictures of the colonies are shown. The data are presented as a histogram of the mean _ SEM of three independent experiments (Student's t-test), **, p<0.01. (F) IFI30 knockdown inhibits cell migration and invasion. Results are representative of three independent experiments. Migrated cells were plotted as the average number of cells per field of view. In the low lane, transwell migration chambers were treated with 10% matrigel, but not in the top lane. **, p<0.01, ***, p<0.001.

As shown in Fig. 1D, IFI30 protein expression was higher especially in triple negative breast cancer and HER-2 positive breast cancer. Consequently, we generated stable IFI30 knockdown cell lines using lentiviral infection in triple negative breast cancer MDA-MB-231 cells and HER-2 positive breast cancer SK-BR-3 cells. Using three stem loop small chains, we found that the IFI30 knockdown effect in No.1 and No.2 stem loop small chains were more pronounced (Fig. 3A and 3B). We next utilized stable transfected MDA-MB-231 cells and SK-BR-3 cells with effective knockdown for functional validation of IFI30. CCK8 experiments showed that knockdown of IFI30 could inhibit the proliferation of MDA-MB-231 cells and SK-BR-3 cells (Fig. 3C and 3D). We obtained the same results as above by clone formation assay with MDA-MB-231 cells (Fig. 3E). After that, we further observed the effect of IFI30 on cell migration and invasion through Transwell assay. The results presented that knockdown of IFI30 could inhibit the migration and invasion of MDA-MB-231 cells (Fig. 3F). In summary, reduced IFI30 expression could lead to less proliferation, migration and invasion of breast cells. It suggested that IFI30 might relate to the development, metastasis and invasion in breast cancer.

### Knockdown of IFI30 could suppress tumor bearing in nude mice

**Figure 4:**
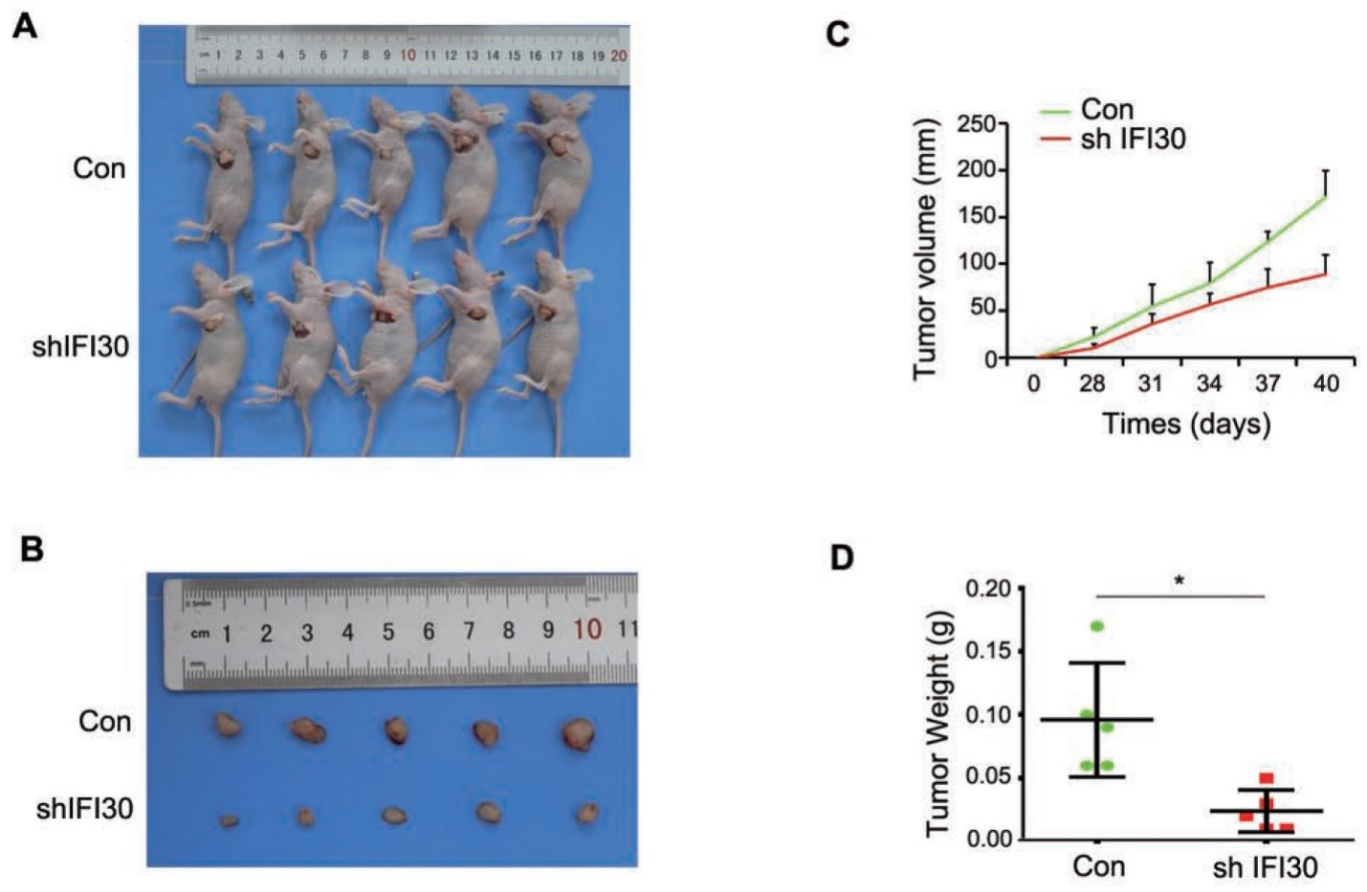
Knockdown of IFI30 could suppress tumor bearing in nude mice. (A) Knockdown of IFI30 suppresses xenograft tumor growth. MDA-MB-231 cells (2×10^6^ cells) transfected with shIFI30 lentivirus and shCtrl (Con) lentivirus were injected subcutaneously into the left flank of nude mice. Mice were imaged at 40 days after injection. (B) Subcutaneous tumors were isolated from nude mice 40 days and photographed. (C) Tumor diameter was measured at the indicated time points, and tumor volume was calculated. The results are presented as the mean SEM of 5 mice per group per time point. (D) Tumor weight in mice from this experiment was measured upon autopsy at Day 40, and the results are presented as a histogram. *, p<0.05.

Our previous results in vitro confirmed that knockdown of IFI30 inhibited the proliferation, migration and invasion of breast cancer cells, and to further confirm the role of IFI30 *in vivo*, we performed subcutaneous tumor bearing experiments in nude mice. MDA-MB-231 cells (2x10^6^ cells) with stable knockdown of IFI30 were injected subcutaneously into nude mice and measurements started after tumors were palpable. Gross images of tumor bearing nude mice (Fig. 4A) and the tumor size (Fig. 4B) were shown. As can be seen from the images, tumors with knockdown of IFI30 were smaller in size than the control group. Tumor production curves revealed that knockdown of IFI30 resulted in inhibition of tumor growth, which was more pronounced with time (Fig. 4C), and mice with IFI30 knockdown had significantly less tumor weight than the controls (Fig. 4D).

### Increased accumulation of LC3-II and p62 suggested impaired autophagy in IFI30 knockdown cells

**Figure 5:**
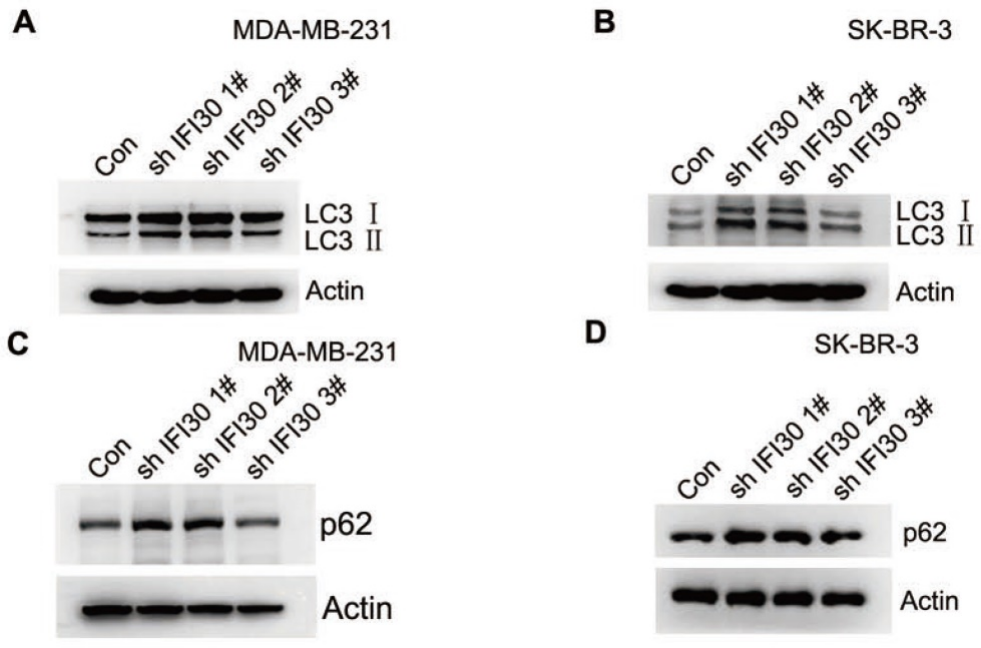
Increased accumulation of LC3-II and p62 suggest impaired autophagy in IFI30 knockdown cells. (A) Western blots of LC3 (LC3-Ⅰ and LC3-II) as indicated by arrows in MDA-MB-231 cells transfected with shIFI30 or shCtrl (Con) lentiviruses as indicated. Actin was used as an internal control. (B) Western blots of LC3 (LC3-Ⅰ and LC3-II) as indicated by arrows in SK-BR-3 cells transfected with shIFI30 or shCtrl (Con) lentiviruses as indicated. Actin was used as an internal control. (C) Western blots of p62 in MDA-MB-231 cells transfected with shIFI30 or shCtrl (Con) lentiviruses as indicated. (D) Western blots of p62 in SK-BR-3 cells transfected with shIFI30 or shCtrl (Con) lentiviruses as indicated.

Within the endosomal/lysosomal compartments, proteins may be trafficked through and tagged for autophagy through the lysosomal degradation pathway. IFI30 resides with the endosomal/lysosomal compartments of cells. In addition, autophagy is closely related to the development of malignant tumors. In our study, we found that knockdown of IFI30 induced the transition of the LC3-Ⅰ to LC3-II in MDA-MB-231 cells and SK-BR-3 cells (Fig. 5A and 5B), which was a significant event in autophagy. According to the autophagy guidelines, LC3 measures cannot distinguish between activation or impairment of the autophagy flux. Therefore, we checked p62 levels and found accumulation of p62 in IFI30 knockdown cells, indicating a potential impairment of autophagy (Fig. 5C and 5D).

## Discussion

With the increasing incidence rate and mortality of breast cancer all over the world, individualized and precise treatment is a hotspot and focus of breast cancer and even various malignant tumors. Therefore, it is urgent to find new molecular targets that respond effectively to breast cancer therapy. Tumor immunity exists in the process of occurrence and development of tumor, which can enhance the ability of killing and recognizing tumor and ensure the tumor clearance. As a result, the immunotherapy of breast cancer has been increasingly used in clinical breast cancer treatment.

In the present study, bioinformatics analysis of TCGA database and CPTAC database indicated that there was a high expression of both mRNA and protein of IFI30 in breast cancer clinical samples. Moreover, significantly higher expression levels of IFI30 were associated with worse OS, RFS and DMFS. We further examined IFI30 protein expression levels in clinical breast cancer samples and obtained the same results, confirming that IFI30 was highly expressed in breast cancer tissues and was associated with poor prognosis.

The function of IFI30 in tumors is still unknown currently. Part of the studies suggested that IFI30 acted as a tumor suppressor in some tumors. The study of Kenneth H. Buetow showed high expression of IFI30 and an active and intact class II MHC antigen presentation pathway was associated with improved melanoma survival 16. Hannah Phipps-Yonas's study indicated that low IFI30 expression was associated with poor patient survival in diffuse large B-cell lymphoma 20. There are also partial studies demonstrated that IFI30 acted to promote tumorigenesis. Study demonstrated that lentivirus mediated IFI30 inhibited the proliferation of human glioma U373MG cell 18. Chen Zhu's study presented that IFI30 was highly expressed in malignant subtypes of glioma, and could reduce the chemosensitivity of glioma cells by activating the IL6-STAT6 signaling pathway 21. In glioma, IFI30 was highly expressed in glioblastomas and gliomas with mesenchymal subtypes or wild-type isocitrate dehydrogenase, which indicated that gliomas were highly malignant and had poor prognosis 22.

It is noteworthy that a study reported that deficiency of IFI30 was associated with poor disease-free survival in breast cancer patients 23, while we obtained different results by bioinformatics and clinical case validation. To further define the function of IFI30, we constructed breast cancer cell lines with stable knockdown of IFI30 using lentiviral infection assay, CCK8 and clone formation assay confirmed that knockdown IFI30 could inhibit the proliferation of breast cancer cells, and transwell assay confirmed that knockdown IFI30 could inhibit the migration and invasion of breast cancer cells. These results confirmed at the cellular level that IFI30 could promote the proliferation, migration and invasion of breast cancer cells. To further investigate the role of IFI30 *in vivo*, we performed tumor bearing experiments in nude mice and obtained the same results. Our results revealed that IFI30 may promote tumorigenesis in breast cancer cells. But the specific mechanism still needs to be elucidated.

Autophagy has many effects on cancer, affecting the occurrence and maintenance of cancer, and regulating the response of cancer to treatment. Beclin1 played an important role in the initiation phase of autophagy. Beclin1, PI3K (hVps34) and ATG14 form a trimer, which continuously collects proteins associated with autophagy, and mediates the beginning of autophagy. Under the action of ATG4, the LC3 precursor is processed into soluble LC3-Ⅰ. LC3-Ⅰ is covalently combined with phosphatidylethanolamine through the action of ATG7, ATG3, and the ATG12-ATG5-ATG16L1 complex to generate LC3-II. The p62 located in the cytoplasm is conjugated to ubiquitin, and then the p62 protein binds to LC3-II protein to obtain some complex, which will eventually be degraded in the lysosome 24. Alterations in autophagy levels caused by mutations of key autophagy genes or aberrant activation of autophagy regulatory factors are related to tumorigenesis (supplementary Table S2). In this regard, the occurrence of cancer is related to a decrease in the level of autophagy, which leads to the accumulation of oncogenes and reactive oxygen species (ROS). On the contrary, during cancer maintenance, the activity of autophagy pathway is often up-regulated. This up-regulation ensures adequate energy supply and helps to survive stress such as hypoxia and metastasis. In the process of anti-cancer treatment, autophagy is enhanced, and cancer cells survive and acquire drug resistance through autophagy. In addition, cancer stem cells (CSCs) seem to rely on autophagy to maintain stem cells 25. Higher levels of autophagic events in tumor cells may also promote lysosomal degradation where IFI30 can influence protein processing by other acidic proteases such as cathepsins. IFI30 maintains the cellular redox state which has implications on autophagy, cellular activation, and proliferation. In the absence of IFI30, mitochondrial membrane potential decreases and autophagy is increased 26. IFI30 expression significantly increased Beclin-1 proteins while decreasing whole LC3 protein 27. In this study, we found significant accumulation of LC3-II in IFI30 knockdown cells, which was consistent with previous reports. In addition, we also found accumulation of p62 in IFI30 knockdown cells, indicating a potential impairment of autophagy.

In conclusion, the present study demonstrated that IFI30 was highly expressed in breast cancer tissues and was associated with poor patient prognosis. Moreover, knockdown of IFI30 inhibited the proliferation, migration and invasion of breast cancer cells and significantly inhibited tumor growth *in vivo*. IFI30 may play a key role in the initiation and progression of human breast cancer and hold promise as a new therapeutic target in breast cancer.

### Ethics Approval and informed consent

This study was approved by the Committee on Medical ethics and Human Clinical Trials of China Medical University. All of the animal experiments were carried out in accordance with the Guidelines for the Care and Use of Laboratory Animals (NIH publication no. 80-23, revised 1996) approved by the Animal Ethics Committee of China Medical University.

## Supplementary Material

Supplementary tables.

## Figures and Tables

**Table 1:** IFI30 expression during breast cancer progression

	IFI30 expression		
Factor	Low (n=39)	High (n=43)	P value
Pathological stage (pStage)			0.173
Stage I	5	2	
Stage II	30	34	
Stage III	4	7	
Lymph node metastasis(pN)			0.199
Yes	18	26	
No	21	17	
Distant metastasis (pM)			0.002**
Yes	4	17	
No	35	26	
ER			0.582
Positive	24	29	
Negative	15	14	
PR			0.908
Positive	24	27	
Negative	15	16	
Her2			0.794
Positive	22	23	
Negative	17	20	

** Indicated statistical significance (p<0.01)

**Table 2:** Univariate and multivariate analysis of IFI30 with regard to OS

	Univariate analysis			Multivariate analysis		
Variable	HR	95% CI	P value	HR	95% CI	P value
*Pathological stage (pStage)*	6.171	2.457-15.497	0.000***	7.939	2.672-23.588	0.000***
*Lymph node metastasis(pN)*	3.338	1.098-10.153	0.034*	1.927	0.614-6.047	0.261
ER	0.741	0.482-1.141	0.174			
PR	0.656	0.394-1.090	0.104			
Her2	1.184	0.863-1.623	0.295			
IFI30	5.63	1.628-19.473	0.006*	6.275	1.701-23.142	0.006**

SE: standard error, HR: hazard ratio, CI: confidence interval. *Indicated statistical significance (p<0.05) ** Indicated statistical significance (p<0.01) *** Indicated statistical significance (p<0.001)

## Abbreviations

IFI30: Interferon γ-inducible protein 30
MHC: major histocompatibility complex
TCGA: The Cancer Genome Atlas
CPTAC: clinical proteomic tumor Analysis consortium
PD1: programmed cell death protein 1
GILT: gamma-interferon-inducible lysosomal thiol reductase
APCs: antigen presenting cells
DCs: dendritic cells
M6P: mannose-6-phosphate
M6PR: M6P receptor
OS: overall survival
DMFS: distant metastasis-free survival
RFS: recurrence free survival
shRNA: small hairpin RNA
HER-2: human epidermal growth factor receptor 2
ROS: reactive oxygen species
CSCs: cancer stem cells
