# Supplementary material for: IFI30 expression predicts patient prognosis in breast cancer and dictates breast cancer cells proliferation via regulating autophagy
Source: Int J Med Sci. 2021 Jul 25;18(14):3342–52. doi: 10.7150/ijms.62870 (PMC8364447; doi:10.7150/ijms.62870)
Supplement: Supplementary file 1 — Supplementary tables. [file ijmsv18p3342s1.pdf]

Table S1. Patient characteristics (IHC)

NO.	Pathological stage	Lymph node metastasis	Distant metastasis	ER	PR	Her2	Survial
1	3	Yes	No	-	-	+	Yes
2	1	No	No	-	-	+	Yes
3	2	Yes	No	+	+	-	Yes
4	3	Yes	Yes	+	+	+	No
5	1	No	No	-	-	+	Yes
6	2	Yes	No	+	+	+	Yes
7	3	Yes	Yes	+	+	-	No
8	2	Yes	No	-	+	-	No
9	1	No	No	+	+	+	Yes
10	3	Yes	Yes	+	-	+	No
11	2	No	No	+	+	+	Yes
12	2	No	No	+	+	-	Yes
13	1	No	No	+	+	-	Yes
14	1	No	No	+	-	-	Yes
15	2	Yes	Yes	-	+	-	Yes
16	1	No	No	+	+	+	Yes
17	3	No	Yes	-	-	+	No
18	2	Yes	No	+	+	+	Yes
19	2	No	No	+	+	+	Yes
20	2	Yes	No	+	+	-	Yes
21	2	No	No	+	+	+	Yes
22	1	No	No	-	-	-	Yes
23	2	No	No	+	+	-	Yes
24	2	Yes	No	+	+	-	Yes
25	2	Yes	No	+	+	-	Yes
26	2	No	No	+	-	+	Yes
27	2	No	No	+	+	+	Yes
28	2	No	No	+	+	-	Yes
29	2	No	No	+	+	-	Yes
30	2	Yes	No	-	+	-	Yes
31	2	Yes	No	+	-	+	Yes
32	2	No	No	+	+	-	Yes
33	2	Yes	Yes	+	-	-	Yes
34	2	No	No	+	+	+	Yes
35	2	No	Yes	-	-	+	Yes
36	2	No	No	+	-	-	Yes
37	2	Yes	Yes	+	+	-	No
38	2	Yes	No	+	+	-	Yes
39	2	Yes	No	+	+	+	Yes

40	3	No	No	-	-	-	Yes
41	2	Yes	Yes	+	+	-	Yes
42	2	Yes	No	+	-	+	Yes
43	2	No	No	-	+	+	Yes
44	2	Yes	No	-	+	-	Yes
45	3	Yes	Yes	-	-	+	No
46	2	Yes	Yes	-	-	+	No
47	2	No	No	+	+	+	Yes
48	2	Yes	Yes	+	+	+	No
49	2	Yes	Yes	-	-	-	No
50	2	Yes	Yes	+	+	-	No
51	2	No	No	-	-	+	Yes
52	2	No	No	+	+	-	Yes
53	2	Yes	No	+	+	-	Yes
54	2	No	Yes	-	-	+	No
55	2	No	No	+	+	-	Yes
56	2	No	No	+	+	+	Yes
57	3	No	No	-	-	+	Yes
58	2	Yes	No	+	-	-	Yes
59	2	Yes	No	+	+	-	Yes
60	2	Yes	No	-	-	-	Yes
61	2	Yes	No	+	+	-	Yes
62	2	No	No	+	+	+	Yes
63	2	Yes	Yes	-	-	+	No
64	2	Yes	No	+	+	-	Yes
65	2	No	No	+	+	+	Yes
66	2	No	No	+	+	+	Yes
67	2	Yes	No	-	-	+	Yes
68	2	Yes	Yes	+	-	+	No
69	2	No	Yes	-	-	+	No
70	2	Yes	No	+	+	+	Yes
71	2	Yes	No	+	+	+	Yes
72	2	Yes	No	-	-	+	Yes
73	2	Yes	No	-	+	-	Yes
74	2	Yes	No	-	+	-	Yes
75	2	No	No	-	-	+	Yes
76	2	Yes	No	+	+	+	Yes
77	3	Yes	No	+	-	-	Yes
78	2	Yes	Yes	+	+	+	No
79	3	Yes	Yes	-	-	+	No
80	2	No	No	+	+	+	Yes
81	2	No	No	-	-	-	Yes
82	3	No	Yes	-	+	+	No

Table S2. Partial autophagy-related protein

Protein	Description
LC3	LC3 is an autophagosomal ortholog of yeast Atg8. A lipidated form of LC3, LC3-II, has been shown to be an autophagosomal marker in mammals. LC3-I is also activated by Atg7, transferred to Atg3, a second E2-like enzyme, and modified to a membrane-bound form, LC3-II. LC3-II is localized to preautophagosomes and autophagosomes, making this protein an autophagosomal marker.
p62/SQSTM1	The p62 protein serves as a link between LC3 and ubiquitinated substrates. p62 becomes incorporated into the completed autophagosome and is degraded in autolysosomes. Inhibition of autophagy correlates with increased levels of p62, suggesting that steady state levels of this protein reflect the autophagic status.
Beclin 1	The mammalian autophagy gene Beclin 1, an ortholog of the Atg6/ vacuolar protein sorting (Vps)-30 protein in yeast. Beclin 1 is important for localization of autophagic proteins to a pre-autophagosomal structure (PAS), depending on interaction with the class III type phosphoinositide 3-kinase (PI3KC3)/Vps34. Together they form the Beclin 1-Vps34-Vps15 core complex. Beclin 1 coordinately regulates the autophagy and membrane trafficking involved in several physiological and pathological processes.
ATGs	Atg12 is first activated by Atg7, then transferred to Atg10 and finally covalently attached to Atg5, a process requiring ATP. The Atg12-Atg5 conjugate localises to autophagosome precursors and dissociates just before or after completion of autophagic-vacuole formation. A second ubiquitin-like modification involving the protein microtubule-associated protein 1 light chain 3 (MAP-LC3 or LC3) is required for completion of autophagosome formation. The cytosolic precursor of LC3 is cleaved at its C terminus by Atg4 to form LC3-I.17 LC3-I is covalently conjugated to phosphatidyl-ethanolamine to form LC3-II, a process requiring the activities of Atg7 and Atg3. LC3-II is specifically targeted to the Atg12-Atg5-associated, elongated autophagosome precursors and remains associated with autophagosomes even after fusion with lysosomes, subsequent to which LC3-II is delipidated and recycled.
